# Cellular and Molecular Mechanisms of Hemorrhagic Shock: Biological Rationale for Individualized Fluid Resuscitation Strategies and Multimodal Monitoring

**DOI:** 10.3390/biomedicines14081678

**Published:** 2026-07-26

**Authors:** Stelian Adrian Ritiu, Sonia Elena Popovici, Marius Papurica, Dorel Sandesc, Adelina Baloi, Daiana Toma, Norbert Wellmann, Petru Bucuras, Claudiu Rafael Barsac, Ovidiu Bedreag

**Affiliations:** 1 Doctoral School, Victor Babes University of Medicine and Pharmacy, 300041 Timisoara, Romania; stelian.ritiu@umft.ro (S.A.R.);; 2 Faculty of Medicine, Victor Babes University of Medicine and Pharmacy, 300041 Timisoara, Romaniabedreag.ovidiu@umft.ro (O.B.); 3 Anaesthesia and Intensive Care Research Center Timisoara, Victor Babes University of Medicine and Pharmacy, 300041 Timisoara, Romania; 4 Clinic for Anaesthesia and Intensive Care, Clinical Municipal Emergency Hospital, 300041 Timisoara, Romania; 5 Clinic of Anaesthesia and Intensive Care, Emergency County Hospital Pius Brinzeu, 300723 Timisoara, Romania; 6 Pulmonology University Clinic, Clinical Hospital of Infectious Diseases and Pneumophthisiology Dr. Victor Babeș, 300310 Timisoara, Romania; 7 Center for Research and Innovation in Personalized Medicine of Respiratory Diseases (CRIPMRD), Victor Babes University of Medicine and Pharmacy, 300041 Timisoara, Romania

**Keywords:** hemorrhagic shock, trauma, cellular mechanisms, glycocalyx, oxidative stress, trauma-induced coagulopathy, fluid resuscitation, multimodal monitoring

## Abstract

Hemorrhagic shock is a leading cause of preventable death following multiple trauma, driven by a cascade of interacting cellular and molecular disturbances that extend well beyond simple volume loss. Acute blood loss initiates tissue hypoperfusion and cellular hypoxia, setting in motion the lethal triad of hypothermia, acidosis, and coagulopathy through several converging pathways: complement activation with excessive C3a and C5a production; neutrophil-mediated tissue injury; NADPH-oxidase-driven reactive oxygen species (ROS) overproduction that overwhelms superoxide dismutase defenses; mitochondrial respiratory chain impairment; dysregulation of the pro-inflammatory cytokine network; and endothelial apoptosis with degradation of the endothelial glycocalyx and disruption of interendothelial junctions, with consequent vascular hyperpermeability. These mechanisms provide the biological rationale for the resuscitation strategy. Each class of fluid acts on these pathways in a distinct way: crystalloids modulate acid–base homeostasis, chloride-mediated renal vasoconstriction, and coagulation factor activity; colloids influence oncotic pressure, endothelial integrity, and microvascular perfusion; and blood products, particularly plasma and whole blood, actively modulate mitochondrial metabolism, endothelial permeability, and pro-apoptotic signaling beyond their volume-expanding role. Translating this biology to the bedside requires a multimodal monitoring framework that converts molecular endpoints into real-time therapeutic targets, integrating lactate and base excess as markers of cellular oxygen debt, dynamic preload indices such as pulse pressure and stroke volume variation, advanced hemodynamic platforms, point-of-care ultrasonography, viscoelastic coagulation testing, and near-infrared spectroscopy of tissue oxygenation. This review synthesizes the biological basis of hemorrhagic shock and its translation into an individualized, goal-directed resuscitation strategy for the critically ill polytrauma patient.

## 1. Introduction

Hemorrhagic shock following multiple trauma represents one of the most complex biological challenges in acute medicine, characterized by a cascade of cellular and molecular disturbances that extends far beyond simple volume loss. Multiple trauma is one of the main causes of disability and death in patients under 40 years of age, with over 150.000 trauma-related deaths reported annually in the United States alone, carrying a substantial social and economic burden [1]. While direct organic injuries, including cerebral trauma, spinal trauma, and injury to vital organs, contribute significantly to early mortality, the most biologically consequential secondary injury is circulatory failure due to hemorrhagic shock. Its impact operates at the cellular and tissue level, triggering a self-amplifying cascade that encompasses systemic inflammatory response syndrome (SIRS), oxidative stress (OS), severe coagulopathy, mitochondrial dysfunction, ventilatory failure, and ultimately multiple organ dysfunction syndrome (MODS), culminating in death if not rapidly and appropriately treated [2,3].

At the molecular level, hemorrhagic shock initiates a series of interrelated pathological processes: the activation of the complement system, the dysregulation of pro-inflammatory cytokine networks, the disruption of redox homeostasis through reactive oxygen species (ROS) overproduction, and the impairment of coagulation factor activity through acidosis and hypothermia. These mechanisms do not occur in isolation; they are mutually reinforcing, forming the biological basis of what has been classically described as the lethal triad of hypothermia, acidosis, and coagulopathy [4]. Understanding these pathways at a mechanistic level is not merely of academic interest; it provides the biological rationale for every therapeutic decision made at the bedside.

Epidemiologically, a consensus analysis of over 28.000 patients from international trauma registries has characterized the polytrauma population: mean age 42.9 years, 72% male, mean Injury Severity Score (ISS) of 30.5, and an overall mortality rate of 18.7%. The consensus defined polytrauma as an ISS greater than 16 combined with an Abbreviated Injury Scale (AIS) score above 2 in at least one of the following physiological variables: hypotension (systolic blood pressure < 90 mmHg), impaired consciousness (Glasgow Coma Scale < 8), acidosis (base excess ≥ 6 mEq/L), coagulopathy (INR > 1.4 or PTT > 40 s), or age above 70 years [5]. Each of these variables reflects a specific biological derangement, underscoring the degree to which clinical definitions of polytrauma are grounded in underlying pathophysiology.

The management of hemorrhagic shock in this population demands not only hemodynamic stabilization but a biologically informed approach to fluid selection, volume titration, and resuscitation timing. The main challenges remain the accurate estimation of blood loss, the selection of the most appropriate resuscitation agent, and the individualization of therapy to the specific biological state of each patient [6]. To address these challenges, international guidelines have incorporated strategies such as permissive hypotension, hemostatic resuscitation, and goal-directed fluid management [7,8,9]. This review examines the cellular and molecular mechanisms underlying hemorrhagic shock, the biological rationale for each class of resuscitation fluid, and the role of multimodal monitoring as the translational interface between biological endpoints and individualized therapeutic decisions.

## 2. Materials and Methods

This narrative review was based on a structured search of the PubMed, Scopus, and Web of Science databases for articles published up to April 2026, using combinations of the search terms “hemorrhagic shock,” “fluid resuscitation,” “crystalloids,” “colloids,” “blood transfusion,” “trauma-induced coagulopathy,” “hemodynamic monitoring,” “viscoelastic testing,” and “near-infrared spectroscopy.” Original research articles, clinical trials, experimental studies, meta-analyses, and relevant international guidelines published in English were considered. Foundational studies establishing key pathophysiological concepts were included regardless of publication date, while priority was given to contemporary evidence for clinical and therapeutic recommendations. Reference lists of included articles were screened manually for additional relevant sources. Studies were selected on the basis of their relevance to the cellular and molecular mechanisms of hemorrhagic shock and the biological rationale for resuscitation and monitoring strategies, with particular emphasis on mechanistic and translational findings.

During the preparation of this manuscript, the authors used Claude (Claude Sonnet 5, Anthropic, PBC, San Francisco, CA, USA) to assist with drafting and language editing of the text and with the preparation of draft versions of the figures. No generative AI tool was used to generate, analyze, or interpret the underlying data, and all data analysis and interpretation were performed by the authors. All authors reviewed and edited the output and take full responsibility for the content of this publication.

## 3. Pathophysiological Mechanisms of Hemorrhagic Shock

The severity of traumatic injuries and their impact on survival are fundamentally linked to acute blood loss, which initiates a cascade of cellular and molecular events centered on tissue hypoperfusion and oxygen deprivation. The resulting imbalance between oxygen delivery and consumption drives a shift from aerobic to anaerobic metabolism, with consequent lactate accumulation, activation of the immune response, dysregulation of the coagulation cascade, and accelerated biosynthesis of pro-inflammatory and pro-oxidative molecules. This multifactorial phenomenon was first described by Moore et al. and subsequently refined, becoming known as the lethal triad of hemorrhagic shock—hypothermia, acidosis, and coagulopathy—each element of which amplifies the others through distinct but interconnected biological pathways [4,10].

### 3.1. Oxygen Delivery, Consumption, and Cellular Hypoxia

The primary molecular consequence of hemorrhagic shock is a critical reduction in oxygen delivery (DO_2_) to tissues. Under physiological conditions, DO_2_ ranges from 1000 to 1250 mL O_2_/min in males and from 925 to 1100 mL O_2_/min in females and is determined by cardiac output multiplied by the arterial oxygen content, itself dependent on hemoglobin concentration and oxygen saturation. A key regulator of tissue oxygen availability is the oxyhemoglobin dissociation (OHD) curve, which describes the relationship between hemoglobin oxygen binding affinity and tissue oxygen unloading. In hemorrhagic shock, the two most important modulators of this curve are pH and temperature. An increase in H^+^ ion concentration shifts the OHD curve to the right (Bohr effect), facilitating oxygen release to hypoxic tissues as a compensatory mechanism, while hypothermia shifts the curve to the left, reducing oxygen delivery at a time when it is most critically needed [11].

Under normal conditions, systemic oxygen consumption (VO_2_) is approximately 250 mL O_2_/min, and the oxygen extraction ratio (O_2_ER = VO_2_/DO_2_) reflects the proportion of delivered oxygen consumed by tissues. Organ-specific O_2_ER values differ substantially (the kidney extracts approximately 15%, the liver 45%, and the myocardium up to 60%), meaning that tissue vulnerability to hypoxia is highly organ-dependent [12]. During hemorrhagic shock, as DO_2_ falls progressively with blood loss, compensatory increases in O_2_ER maintain VO_2_ constant up to a blood volume loss of approximately 30%. Beyond this threshold, O_2_ER reaches its physiological ceiling of 60–70%, after which VO_2_ becomes flow-dependent, a critical inflection point that marks the transition from compensated to decompensated shock, and the onset of irreversible cellular injury [11,13]. Hemorrhagic shock is graded into four classes according to the volume of blood lost. Class I involves up to 15% loss (approximately 750 mL) with minimal physiological change; Class II, 15 to 30% (750 to 1500 mL), produces tachycardia, tachypnoea, and a narrowed pulse pressure; Class III, 30 to 40% (1500 to 2000 mL), causes overt hypotension, marked tachycardia, and altered mental status; and Class IV, greater than 40% (over 2000 mL), is immediately life-threatening, with profound hypotension and negligible urine output. 

### 3.2. Innate Immune Activation and Neutrophil-Mediated Injury

Shortly after traumatic injury, molecular danger signals, including damage-associated molecular patterns (DAMPs) released from injured cells, activate both cell-autonomous and non-self-mediated inflammatory pathways [5]. A central effector of the early post-traumatic inflammatory response is neutrophil activation. Primed neutrophils undergo rapid tissue infiltration, releasing proteases, ROS, and neutrophil extracellular traps (NETs) that amplify local and systemic tissue injury [14].

The gene expression profile of post-traumatic neutrophils has been characterized in detail by Bogner-Flatz et al. [15], who quantified the expression of *IL-1*, *c-JUN*, *BCL2a*, *c-FOS*, *TIMP-1*, *MMP-9*, *ETS-2*, and *MIP-1* in 40 trauma patients (mean ISS 36 ± 14) at 6, 12, 24, 48, and 72 h after injury. The study demonstrated significant correlations between the expression of *BCL2a*, *MMP-9*, *TIMP-1*, and *ETS-2* and 90-day survival rates, and between the expression of *IL-1*, *MIP-1*, *MMP-9*, and *BCL2a* and the need for blood transfusion, establishing a direct link between neutrophil-driven molecular activity and clinically relevant outcomes [15].

### 3.3. Complement System Activation

Complement activation is an early and potent amplifier of the post-traumatic inflammatory response. Tissue injury triggers the classical, lectin, and alternative pathways of complement, generating highly reactive anaphylatoxins and opsonins. Of particular importance is the underexpression of C1qrs and C3b opsonin activity, which leads to the excessive production of C3a and C5a, which are potent anaphylatoxins that drive mast cell degranulation, neutrophil recruitment, and endothelial activation [16,17]. The downstream consequence is a bioenergetic crisis at the level of the mitochondrion. Sustained hypoperfusion deprives the electron transport chain of the oxygen that serves as its terminal electron acceptor, so that oxidative phosphorylation fails and the cell reverts to inefficient anaerobic glycolysis with progressive ATP depletion and lactate accumulation. Complement-activated cytokines and ROS generated during shock compound this by directly impairing the respiratory chain complexes and uncoupling oxidative phosphorylation. As intracellular calcium and reactive oxygen species rise, the mitochondrial permeability transition pore opens, dissipating the mitochondrial membrane potential and releasing cytochrome c, a pivotal event that commits the cell to death by apoptosis or, when the insult is severe and ATP is exhausted, by necrosis. Reperfusion paradoxically intensifies this injury, because the abrupt reintroduction of oxygen to a reduced respiratory chain produces a burst of reactive oxygen species. Mitigation of this mitochondrial injury remains largely indirect: the principal intervention is the timely restoration of perfusion and oxygen delivery before pore opening becomes irreversible, which is the bioenergetic rationale for early hemostatic resuscitation, whereas directly mitochondria-targeted strategies, such as permeability transition pore inhibition and mitochondria-targeted antioxidants, remain experimental and are not yet part of clinical practice [18].

### 3.4. Oxidative Stress and Redox Dysregulation

Hemorrhagic shock is associated with a profound disruption of redox homeostasis, characterized by the overproduction of reactive oxygen species (ROS) that overwhelms endogenous antioxidant defenses [19]. One of the primary sources of ROS in this context is the activation of NADPH-oxidase at the cellular membrane, driven by complement factors and arachidonic acid pathway intermediates. The body’s enzymatic antioxidant response, mediated by cytosolic superoxide dismutase (SOD1), extracellular SOD3, and mitochondrial SOD2, catalyzes the reduction in superoxide anion (O_2_^−^) to hydrogen peroxide. However, once these enzymatic resources are exhausted, ROS accumulate and interact with myeloperoxidase to generate additional reactive species, including the highly damaging hydroxyl radical (OH^−^). These radicals attack membrane lipids and proteins, causing endothelial and parenchymal cell destruction, interfering with nitric oxide (NO) signaling pathways, and generating nitrogen-free radical species that further amplify the pro-inflammatory milieu [20]. These redox disturbances have been characterized directly in the critically ill polytrauma population, including in our own institutional cohorts. Prospective and retrospective studies from our group have documented elevated OS biomarkers, among them markers of lipid peroxidation, in polytrauma patients, and have shown that targeted antioxidant therapy correlates with reductions in these markers of molecular damage and with improvements in clinical course [6,21]. These observations connect the redox mechanisms described here to measurable, and potentially modifiable, endpoints at the bedside. NF-κB is a central transcriptional regulator of the inflammatory response, controlling the expression of pro-inflammatory cytokines, chemokines, and adhesion molecules that drive the systemic inflammation seen in hemorrhagic shock [22]. Modulation of this pathway has therefore been proposed as a molecular target through which resuscitation strategies may attenuate the post-traumatic inflammatory cascade.

### 3.5. Cytokine Cascade and Systemic Inflammatory Response

The oxidative and complement-driven molecular events described above converge on a coordinated cytokine response. Within two hours of traumatic injury, IL-1β and TNF-α are the first mediators to show marked upregulation. Their biosynthesis subsequently drives the production of IL-6 and IL-8, which activate neutrophils and impair macrophage function, and of IL-12, which together with IL-8 modulates interferon-γ and high mobility group protein-1 (HMG-1) expression, further sustaining the inflammatory cascade [17].

The clinical relevance of this cytokine response is supported by evidence linking circulating proinflammatory cytokine levels to outcome in critical illness. A systematic review and meta-analysis comparing cytokine concentrations in patients with sepsis and healthy volunteers confirmed that IL-6 and TNF-α are markedly elevated in the systemic inflammatory state and identified an association between TNF-α levels and sepsis-related mortality. Although derived predominantly from sepsis cohorts, these findings are mechanistically relevant to hemorrhagic shock, in which the same cytokine networks are activated, and they support the use of IL-6 and TNF-α as candidate biomarkers of inflammatory severity and prognosis [23]. It should be emphasized, however, that this cytokine response is not intrinsically pathological. The early elevation of IL-1β, TNF-α, and IL-6 is an appropriate, evolutionarily conserved innate response to tissue injury and hypoperfusion, mobilizing host defense and initiating repair. It becomes maladaptive only when it is excessive, loses its local compartmentalization, and becomes systemic and self-sustaining, at which point it drives the systemic inflammatory response and contributes to organ injury. The distinction is quantitative as well as qualitative: absolute circulating cytokine concentrations in hemorrhagic shock are generally lower than those reached in established sepsis, and the term dysregulation is therefore best applied to the loss of this compartmentalization and proportionality rather than implying that the response is aberrant from its inception [24].

**Figure 1:**
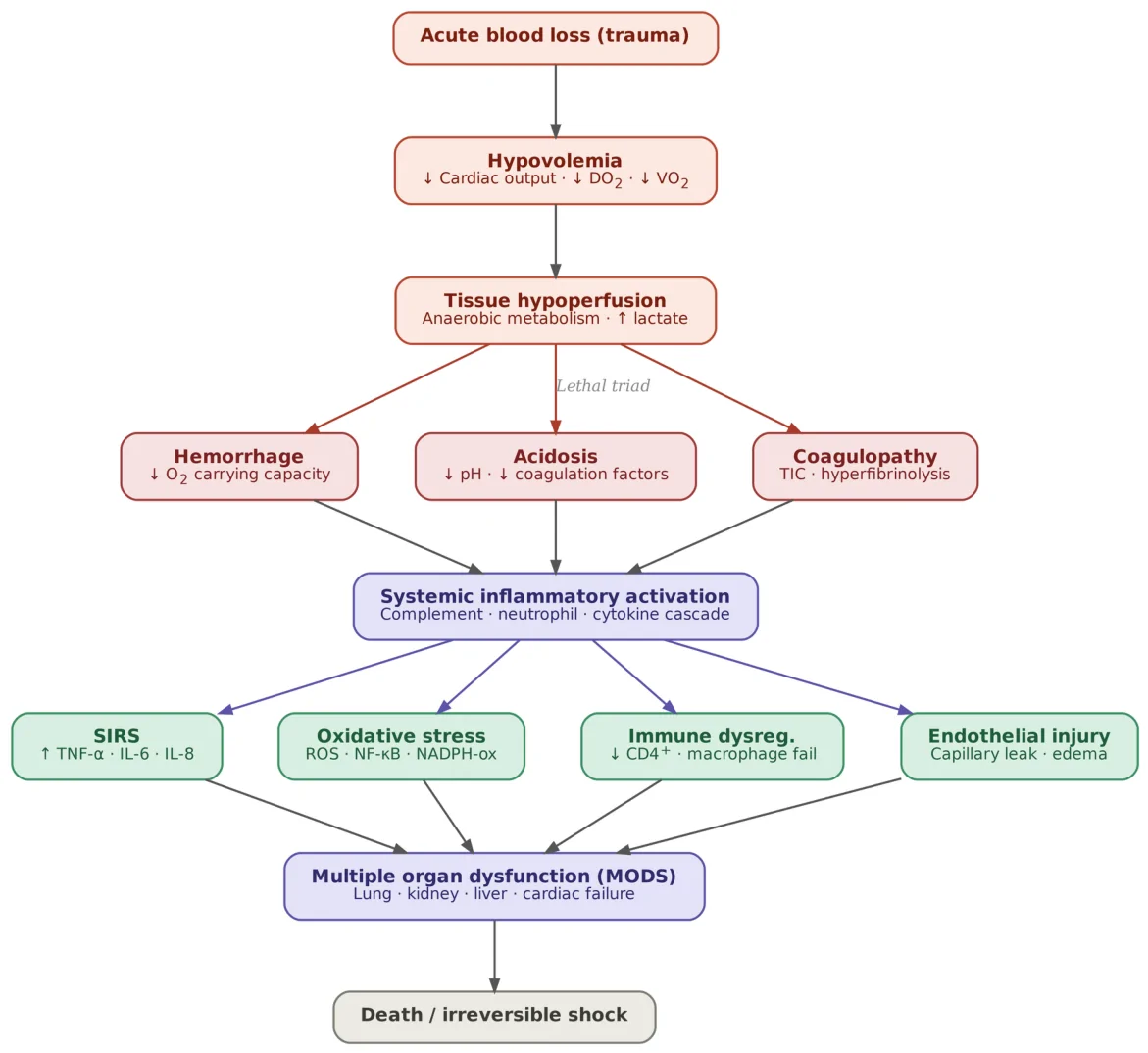
The pathophysiological cascade of hemorrhagic shock, from acute blood loss to multiple organ dysfunction and irreversible shock. Box colours denote successive phases of the cascade: initial haemodynamic insult (peach), the lethal triad (red), systemic inflammatory nodes (lavender), and downstream effector mechanisms (green); the grey box marks the terminal outcome. Arrows indicate the direction of causal progression; arrow colour reflects the originating phase and carries no additional meaning. Abbreviations: DO_2_ = oxygen delivery; VO_2_ = oxygen consumption; TIC = trauma-induced coagulopathy; TNF-α = tumour necrosis factor alpha; IL = interleukin; ROS = reactive oxygen species; NF-κB = nuclear factor kappa B; NADPH-ox = NADPH oxidase; CD4^+^ = CD4-positive T lymphocyte; SIRS = systemic inflammatory response syndrome; MODS = multiple organ dysfunction syndrome.

The complex pathophysiological cascade triggered by acute blood loss, from initial hypovolemia and tissue hypoperfusion through the lethal triad of hypothermia, acidosis, and coagulopathy, to downstream systemic inflammatory activation and ultimately multiple organ dysfunction, is summarized in Figure 1.

### 3.6. The Endothelial Glycocalyx and Vascular Barrier Dysfunction

A unifying element underlying the endothelial dysfunction described above is the degradation of the endothelial glycocalyx, a gel-like layer of membrane-bound proteoglycans, glycoproteins, and glycosaminoglycans (principally syndecan-1, heparan sulfate, and hyaluronan) that lines the luminal surface of the vascular endothelium and regulates permeability, leukocyte adhesion, and coagulation [25]. In hemorrhagic shock, the combination of hypoperfusion, ischemia-reperfusion injury, sympathoadrenal activation, and the inflammatory mediators already discussed drives rapid enzymatic and oxidative shedding of this layer, a process termed endotheliopathy of trauma [26]. Circulating syndecan-1 has emerged as a quantifiable biomarker of glycocalyx degradation, with elevated plasma levels correlating with coagulopathy, increased transfusion requirements, and mortality in trauma patients [27].

The biological consequences of glycocalyx breakdown are central to the pathophysiology of fluid extravasation. Loss of this barrier exposes endothelial adhesion molecules, increases capillary permeability, and permits the interstitial leakage of fluid and protein that manifests clinically as tissue edema and the “third-space” losses characteristic of shock. Critically, this mechanism reframes the classical Starling model of fluid exchange: the revised Starling principle recognizes the subglycocalyx space, rather than the interstitial colloid osmotic pressure, as the key determinant of transcapillary fluid flux [28]. It should be noted that the precise behavior of the subglycocalyx space remains debated; this uncertainty does not, however, alter the practical conclusion, since the revised Starling forces differ from the classical model by an approximately constant term, and the same therapeutic principles therefore apply. On the other hand, this has direct therapeutic implications, as aggressive crystalloid administration may itself accelerate glycocalyx shedding and worsen the very capillary leak it is intended to correct, whereas plasma and whole blood appear to exert a protective, glycocalyx-restoring effect, providing a molecular rationale for plasma-first resuscitation strategies [29].

### 3.7. Host Genetic and Genomic Determinants of the Response to Injury

The observation that patients with comparable injury severity and physiology can follow strikingly different clinical trajectories, from rapid recovery to progressive organ failure, points to a substantial host-dependent component in the response to trauma and hemorrhagic shock. Part of this interindividual variability is genetically determined and operates through two broad axes relevant to the mechanisms described above: the intensity of immune reactivity and the robustness of cellular defense against oxidative injury.

Immune reactivity is modulated by common polymorphisms in the genes encoding cytokines and innate immune receptors. In a cohort of severely injured patients with traumatic hemorrhagic shock, single-nucleotide polymorphisms across thirteen cytokine genes, including *TNF-α* (−308), *IL-1β* (−511), *IL-6* (+565), and *IL-10* (−1082), were significantly associated with the subsequent development of sepsis, organ dysfunction, and mortality, indicating that the genetically set magnitude of the inflammatory response helps determine outcome [30]. Variation in the pattern-recognition machinery is similarly consequential: functional polymorphisms in Toll-like receptor 4 (*TLR4*) and in the *TNF-α* promoter alter the innate response to damage-associated molecular patterns and endotoxin and have been linked to the risk of severe sepsis and post-traumatic organ failure [31]. These variants provide a mechanistic basis for the clinical heterogeneity of the systemic inflammatory response and identify patients who are constitutionally predisposed to an exaggerated or, conversely, an inadequate immune reaction.

Cellular defense against the oxidative burst of shock and reperfusion is likewise under genetic control, principally through the Nrf2–Keap1–antioxidant response element pathway, the master transcriptional regulator of the endogenous antioxidant program that governs the superoxide dismutase and glutathione systems discussed in Section 3.4. A key Nrf2 effector is heme oxygenase-1 (*HO-1*, encoded by *HMOX1*), an inducible enzyme that degrades pro-oxidant free heme and confers broad cytoprotection. Nrf2 activation and *HO-1* induction protect against the oxidative tissue injury of hemorrhagic shock and ischemia-reperfusion, whereas Nrf2 deficiency exacerbates it, and functional variation in the *HMOX1* promoter that sets the level of *HO-1* inducibility has been associated with differing susceptibility to oxidative and ischemia-reperfusion injury [32,33]. The capacity to mount this antioxidant defense therefore represents a genetically determined reserve that shapes whether a given oxidative insult is contained or becomes a driver of cellular death.

Beyond individual variants, severe injury elicits a coordinated, genome-wide reprogramming of the circulating leukocyte transcriptome, and it is the pattern and speed of resolution of this response, rather than any single gene, that most closely tracks with recovery as opposed to a complicated course culminating in persistent immunosuppression and chronic critical illness [34]. Recognizing this genetic and genomic dimension reframes individualized resuscitation as a strategy that must ultimately account not only for the measured physiology of the patient but for the host’s constitutional capacity to defend against, and recover from, cellular injury.

## 4. Fluid Resuscitation Formulas: Current Evidence and Updates

The biological consequences of fluid administration in hemorrhagic shock extend well beyond simple volume replacement. Each class of resuscitation fluid interacts with the cellular and molecular environment of the shocked patient in distinct ways: modulating acid–base homeostasis, electrolyte balance, immune cell function, coagulation factor activity, and organ perfusion at the tissue level. Understanding the biochemical properties of these agents and their downstream biological effects is therefore essential for rational, individualized fluid selection. The following subsections examine the biological rationale and clinical evidence for crystalloids, colloids, and blood products in the context of hemorrhagic shock resuscitation.

### 4.1. Crystalloids: Composition and Biological Effects

In daily practice more than one type of fluid is used for volemic resuscitation, among which are crystalloids, colloids, and blood products. From a chemical point of view, crystalloids are aqueous solutions to which inorganic ions are added, as well as organic molecules with low molecular weights. During the last few decades, new formulas for crystalloid solutions have been developed, with different concentrations of potassium, magnesium, calcium, and other inorganic ions such as lactate and acetate. The composition of these solutions is extremely important, as it has a direct impact on cell and tissue function, as well as an indirect impact on the functionality of certain organs, as well as certain physiological pathways such as the coagulation cascade [35].

After administration, normal saline is redistributed in a short period of time between the compartments of the extracellular space. Greenfield et al. [36] have evaluated the effects intravenous normal saline administration has on hematocrit levels in 28 healthy volunteers. The participants in the study received 10, 20 and 30 mL/kg NaCl 0.9% as a bolus, at a rate of 115 ± 4 mL/min, followed by a continuous infusion at 1–5 mL/kg/h. Immediately after the bolus administration, they determined the blood hematocrit and compared it to the starting value, with the measurements showing statistically significant differences. The administration of a 10 mL/kg bolus of normal saline led to a decrease in hematocrit by 4.5 ± 0.6 (*p* < 0.001), a 20 mL/kg bolus led to a decrease by 6.1 ± 0.4 (*p* < 0.01), while in the group that received a 30 mL/kg bolus of normal saline the hematocrit dropped by 6.3 ± 0.6 (*p* < 0.01). Twenty minutes after the infusion was started, hematocrit was measured again and compared to the value after the bolus dose, showing an increase in all three patient groups with 1.5 ± 0.8 (*p* = 0.03), 2.4 ± 0.4 (*p* = 0.004), and 2.3 ± 0.7 (*p* = 0.005) respectively. This study demonstrated that normal saline solution will diffuse in less than 20 min in the extravascular space when administered as an intravenous bolus [36]. A similar study was carried out by Mullins et al. [37] in 126 patents that were administered 2 L of normal saline over 2 h, one day before surgery. They compared hemoglobin before and after the infusion and calculated the change in blood volume. The differences before and after the infusion were 1.06 ± 0.06, which shows a 18–24% retention of the infused solution [37]. Other studies have shown that up to 60% of the volume of the administered fluid will stay in the body even longer than 6 h, and approximately 13% will stay in the intravascular space [38]. Hahn et al. [39] have shown in their study that the distribution and elimination of crystalloid solutions is delayed in surgical patients in comparison to healthy volunteers [39]. An important caveat applies to these observations: volunteer studies were performed in healthy subjects with an intact endothelial glycocalyx. In trauma and hemorrhagic shock, where the glycocalyx is already degraded and capillary permeability increased, crystalloid extravasation from the intravascular space is likely to be substantially faster and more pronounced than these figures suggest, further limiting the volume-expanding efficacy of crystalloids in precisely the setting where it is most needed.

### 4.2. Hyperchloremic Acidosis and Balanced Solutions

For a long time, fluid resuscitation was based on high amounts of normal saline (NaCl 0.9%), but numerous studies concluded that it leads to hyperchloremic metabolic acidosis. Waters et al. [40] directly compared the clinical consequences of normal saline and lactated Ringer’s solution in a double-blinded randomized trial of 66 patients undergoing aortic reconstructive surgery, with 33 patients allocated to each solution under standardized anesthetic and fluid management [40]. Patients receiving normal saline developed hyperchloremic metabolic acidosis and consequently required substantially more bicarbonate therapy to correct a base deficit greater than −5 mEq/L (30 ± 62 mL versus 4 ± 16 mL in the lactated Ringer’s group). Notably, the acidosis was also associated with greater transfusion requirements: the normal saline group received a larger volume of platelet transfusion (478 ± 302 mL versus 223 ± 24 mL) and significantly more blood products overall (*p* = 0.02). No differences were observed between the groups in duration of mechanical ventilation, intensive care unit stay, hospital stay, or incidence of complications. The authors concluded that although saline-induced acidosis did not alter these clinical outcomes, it necessitated greater bicarbonate administration and was associated with increased blood product use—findings particularly relevant to procedures involving extensive blood loss and large-volume fluid resuscitation, such as the management of hemorrhagic shock. These findings can be explained by the fact that the intravenous administration of normal saline increases the extracellular volume, which in turn dilutes the buffer systems, especially the bicarbonate buffer system; this phenomenon is called dilution acidosis [40]. Gattinoni et al. [41] simulated this dilution phenomenon by infusion of hydrogen peroxide, normal saline, and Lactated Ringer’s solution. After this study, they concluded that the pH of the system is only modified when CO_2_ is infused in the solution, which in turn will change the pK constant following the dissociation of CO_2_ and bicarbonate [41].

Scheingraber et al. [42] carried out a randomized study on 12 patients who were allocated to two study groups—one receiving normal saline and the other Ringer’s lactate with equivalent doses of 30 mL/kg/h. They measured pH, serum sodium, potassium, chloride, and lactate, as well as the partial pressure of carbon dioxide. In the case of patients receiving normal saline, a hyperchloremic metabolic acidosis could be seen (104 mmol/L vs. 115 mmol/L, pH 7.4 vs. pH 7.2), as well as a decrease in base excess [42].

Acidosis induced by high-chloride crystalloids can be mitigated by using balanced solutions containing anions that the body can metabolize, such as lactate, acetate, citrate, and malate. Widely used balanced crystalloids include Sterofundin (B. Braun Melsungen AG, Melsungen, Germany), Plasma-Lyte (Baxter International Inc., Deerfield, IL, USA), Hartmann’s solution, lactated Ringer’s, and acetated Ringer’s [43]. One trade-off of balanced solutions is a slight reduction in plasma osmolarity—lactated Ringer’s has a calculated osmolarity of 273 mOsmol/L but a measured osmolarity of approximately 254 mOsmol/kg—and the intravenous administration of relatively hypotonic solutions can promote cerebral edema or increased diuresis. Consistent with this, Reid et al. [44] demonstrated a doubling of diuresis six hours after infusion of Hartmann’s solution compared with normal saline [44]. One implication of this finding is that normal saline is retained within the circulation somewhat longer than Hartmann’s solution, which may be advantageous when sustained intravascular volume is the priority or disadvantageous when it contributes to fluid accumulation, depending on the clinical circumstance.

### 4.3. Renal and Coagulation Effects of Crystalloids

In polytrauma patients, one of the main organs affected by the metabolic imbalances is the kidney. The kidney can suffer from a direct traumatic injury or can be injured indirectly through massive blood loss leading to hypoperfusion, through delayed resuscitation or rhabdomyolysis. Up to 40% of trauma patients can develop acute kidney injury in the first phase of treatment, but in this case, it is a reversible dysfunction if correct and optimal hemodynamic management and fluid resuscitation occur [45,46]. However, kidney injury can also be precipitated by the type and volume of solutions used for fluid resuscitation.

Ramesh et al. [47], in a study on fluid resuscitation in trauma, concluded that the administration of a high volume of fluids can be associated with the development of hypoxia, acidosis, coagulopathy, dilution-induced hyperfibrinolysis, and multiple organ failure [47]. Recent guidelines recommend adopting restrictive fluid management in the first phase of treatment and avoiding the use of lactated Ringer’s solution in traumatic brain injury, especially because of the correlations reported between increased incidence of cerebral edema and the infusion of large volumes of lactated Ringer’s. A scoping review of lactate- versus acetate-buffered crystalloid solutions has summarized the evidence that acetate-buffered solutions may offer more favorable acid–base and metabolic profiles compared with normal saline, particularly in patients with impaired hepatic lactate metabolism [48]. Regarding the use of artificial colloids, European guidelines suggest a restrictive use, especially due to their adverse effects on homeostatic mechanisms. Early trials suggested a potential role for modern tetrastarch (HES 130/0.4) in trauma resuscitation: in the FIRST trial, HES reduced fluid requirements in penetrating trauma compared with saline, although in blunt trauma it was associated with significantly greater blood product use [49]. However, subsequent large randomized trials in the critically ill, most notably the CHEST trial, demonstrated no mortality benefit and an increased need for renal-replacement therapy with HES [50]. A randomized controlled trial from Duncan et al. shows consistent signals of renal and hemostatic harm without a clear beneficial effect in any subgroup, and contemporary trials in trauma and surgical populations do not demonstrate clear benefit [51]. On this basis, regulatory restrictions have been imposed, and HES is no longer recommended for resuscitation in trauma or critical illness.

Beyond the colloids, the chloride load of unbalanced crystalloids is itself a determinant of renal injury. The supraphysiological chloride concentration of 0.9% saline promotes renal vasoconstriction and a reduction in renal cortical perfusion, mediated by tubuloglomerular feedback. In a randomized, double-blind study in healthy volunteers, administration of saline reduced renal cortical tissue perfusion and renal blood flow velocity compared with a balanced crystalloid (Plasma-Lyte), as assessed by magnetic resonance imaging [52]. These hemodynamic effects translate into clinically meaningful outcomes: large randomized evidence from the SMART trial demonstrated that balanced crystalloids reduced the incidence of major adverse kidney events compared with saline in critically ill adults [53]. The high chloride content of normal saline, rather than its sodium or volume, therefore appears to be the principal driver of its adverse renal effects.

### 4.4. Balanced Crystalloids Versus Saline: Comparative Evidence and Clinical Outcomes

Because each crystalloid carries a distinct electrolyte and osmolarity profile, both its distribution between fluid compartments and its metabolic effects differ; the measured plasma osmolarity of approximately 288 mOsmol/kg H_2_O, closely matching the calculated 291 mOsmol/L, is the physiological reference against which these solutions are judged [54]. Two questions follow: whether balanced solutions improve patient-centered outcomes relative to saline, and how balanced solutions differ among themselves.

At the level of clinical outcomes, large randomized trials have been broadly concordant. Balanced crystalloids consistently reduce hyperchloremia and, in some cohorts, major adverse kidney events relative to saline, but the largest trials do not translate this into a clear survival or renal-replacement benefit in unselected populations. The SMART trial showed a modest reduction in major adverse kidney events in critically ill adults, whereas the SALT-ED trial in non-critically ill adults was neutral for its primary outcome of hospital-free days, and the PLUS trial of 5037 patients found no difference in 90-day mortality (21.8% vs. 22.0%) or acute kidney injury. Together these data justify balanced crystalloids as the physiologically preferable first-line choice while indicating that their incremental outcome advantage over saline is small at the population level. This near-equivalence in outcomes has a practical corollary: because balanced solutions such as Plasma-Lyte cost appreciably more than saline while conferring only a marginal outcome advantage, cost and local availability legitimately influence fluid selection, and saline retains a defined role rather than being obsolete. The clinically important exception is traumatic brain injury, in which the relative hypotonicity of balanced solutions may aggravate cerebral edema, and saline remains preferable [43,55,56].

Among the balanced solutions themselves, the differences are physiological rather than outcome-defining. A meta-analysis of 24 trials found that Plasma-Lyte produced the smallest rise in plasma chloride and lactate of any balanced crystalloid [56], and randomized comparisons of saline with acetate- and malate-buffered solutions (Sterofundin ISO)(B. Braun Melsungen AG, Melsungen, Germany) in brain-injured and neurosurgical patients consistently show better preservation of base excess, bicarbonate, and chloride homeostasis with the balanced solution [57,58]. Buffer choice matters mainly in specific contexts: lactate- and acetate-buffered Ringer’s solutions are broadly equivalent in acid–base and hemodynamic terms in general surgical patients [59], while acetate- or bicarbonate-buffered solutions are theoretically preferable when hepatic lactate metabolism is impaired. In hemorrhagic shock specifically, a bicarbonate-buffered Ringer’s solution accelerated correction of lactate and base excess and was associated with shorter mechanical ventilation and intensive care stay and a lower incidence of acute respiratory distress syndrome than saline, although 28-day survival was unchanged [60]. The composition, adverse-effect, and guideline profiles of these solutions are summarized in Table 1.

### 4.5. Immune and Apoptotic Effects of Resuscitation Fluids

Resuscitation fluids are not immunologically inert. Beyond their effects on acid–base and electrolyte balance, their composition and tonicity modulate immune cell activation and apoptotic signaling, and these properties have been proposed as an additional basis for fluid selection. Experimental work supports three observations. First, tonicity itself is immunomodulatory: in a hemorrhagic shock model, hypertonic saline attenuated the disturbance of CD4+ T-lymphocyte populations relative to isotonic saline [61], consistent with broader evidence that hyperosmolarity dampens neutrophil activation and pro-inflammatory signaling. Second, the buffer anion matters independently of the fluid class: in a rat hemorrhage model, racemic lactated Ringer’s induced hepatic apoptosis, whereas removing the D-lactate isomer or substituting beta-hydroxybutyrate or pyruvate reduced apoptosis and increased the anti-apoptotic protein bcl-2, an effect independent of hepatic energy status [62]. Third, targeting the cellular pathways that couple ischemia to inflammation can be organ-protective: inhibition of the sodium–hydrogen exchanger (NHE-1) during resuscitation reduced hepatic inflammatory markers and neutrophil infiltration and lowered plasma alanine aminotransferase, a marker of hepatocellular injury, indicating attenuation of liver cell death rather than a change in a laboratory value alone [62].

The clinical significance of these immune effects, however, must be judged against outcome data, and here the evidence is sobering. The most extensively studied immunomodulatory fluid, hypertonic saline, was advanced precisely on the rationale that modulating the inflammatory response would reduce secondary organ injury, yet the large randomized Resuscitation Outcomes Consortium trials found no survival benefit in traumatic hypovolemic shock and no improvement in survival or neurologic outcome in traumatic brain injury, and both were stopped early for futility [63,64]. This dissociation also answers a natural question: if hypertonic saline is beneficial, why not isotonic 0.9% saline? The immunomodulatory effect is a property of hypertonicity, the transient hyperosmolar state that alters immune cell volume and signaling, and not of sodium chloride as such. Isotonic saline confers no comparable effect and, through its chloride load and consequent acidosis, is, if anything, pro-inflammatory. The immune and apoptotic effects of resuscitation fluids are therefore biologically real and mechanistically informative, but they have not translated into outcome benefits attributable to immunomodulation. They are best regarded as a secondary consideration in fluid selection, subordinate to the acid–base, renal, and coagulation effects discussed above and, in hemorrhage specifically, to the primacy of blood products.

### 4.6. Colloids: Albumin, HES, and Gelatins

Colloids expand the intravascular volume more efficiently than crystalloids, because their oncotic pressure retains fluid within the vascular space, so that for a given hemodynamic effect they require a smaller infused volume [65].

Whether this efficiency benefits the patient depends heavily on the specific colloid and the clinical context.

Albumin is the most extensively studied colloid and illustrates the point. It reaches resuscitation targets with less volume than crystalloid, but this advantage comes at substantially higher cost and, critically, without a general survival benefit: in the SAFE trial of nearly 7000 critically ill adults, 4% albumin and saline produced identical mortality and organ-support outcomes [66]. Experimentally, albumin does improve the microcirculation, reducing leukocyte adhesion and enhancing mesenteric perfusion in hemorrhagic shock [67], which explains its physiological appeal. The clinical picture, however, is decisively context-dependent: in the traumatic brain injury subgroup of the same program, albumin was associated with significantly higher mortality and is now contraindicated in that setting [68], whereas in sepsis the signal was toward benefit. Albumin is therefore best reserved for selected indications such as sepsis or hypoalbuminemia, rather than used routinely in undifferentiated hemorrhagic shock, and it does not substitute for blood products when hemorrhage is the primary problem. Hydroxyethyl starches (HES) are available in multiple semisynthetic formulations differing in concentration, molecular weight, and degree of molar substitution [69].

Their clinical trajectory, however, can now be summarized simply. Because HES accumulates in the reticuloendothelial tissues and kidney and causes osmotic nephrosis, both randomized and experimental studies have consistently demonstrated an increased incidence of acute kidney injury, a greater need for renal replacement therapy, and tubular damage relative to crystalloids or gelatins, without any offsetting benefit [70,71,72].

Consistent with the trial-level evidence and regulatory restrictions discussed in Section 4.3, HES has been suspended for use in critically ill and septic patients and has largely disappeared from trauma resuscitation, having been little used in North America since around 2010. HES is not recommended in hemorrhagic shock. Gelatins, the remaining semisynthetic colloid, rest on little trauma-specific evidence and carry risks of anaphylaxis and, at high volumes, coagulopathy; they are at most an alternative when albumin is unavailable.

Taken together, colloids expand plasma volume and improve cardiac preload more efficiently than crystalloids, an effect demonstrable even intraoperatively [73], but this hemodynamic efficiency has not translated into better patient outcomes, and two of the three classes carry demonstrable harm (HES in general, albumin in traumatic brain injury). In the resuscitation of hemorrhagic shock specifically, colloids have no first-line role in hemorrhagic shock, where crystalloids serve only as a bridge and blood products are the definitive resuscitation, as detailed in Section 6. The comparative composition, cost, adverse-effect, and guideline profiles of these agents are summarized in Table 1.

### 4.7. Permissive Hypotension and Tissue Oxygenation

Tissue oxygenation can have a significant impact on clinical outcome in patients with hemorrhagic shock, being directly related to hemodynamic status, and especially to tissue perfusion pressure, blood flow, and cardiac output. Kurita et al. [74], in an animal experimental study, analyzed the impact hemorrhagic shock has on gas exchange. Hemorrhagic shock was induced through the aspiration of 600 mL of blood, followed by the infusion of 600 mL of HES. After each blood aspiration, apnea was induced and desaturation time to SpO_2_ < 70% was measured. The difference in desaturation time between baseline and the first blood loss was 11.2 s (*p* = 0.0052), and 16 s after the second blood loss (*p* < 0.0001). Oxygen consumption decreased during hypovolemia, and DO_2_ was recovered following the normalization of perfusion pressure [74]. However, aggressive fluid resuscitation in trauma is known to be associated with a series of complications that have a significant impact on mortality. This is the reason why a new concept appeared, meaning the maintenance of permissive hypotension that is able to reduce bleeding, maintain adequate organ perfusion, and reduce mortality [75]. Tran et al. [76], following a meta-analysis including 722 papers and 1158 patients, concluded that permissive hypotension brings further benefits in patient survival compared with conventional fluid resuscitation in the case of hemorrhagic shock. Moreover, it reduces the need for blood transfusion and improves coagulation status, decreasing bleeding [76]. Another meta-analysis by Owattanapanich et al. [77] identified a decrease in mortality rates in patients in whom fluid resuscitation was carried out while maintaining a degree of permissive hypotension. No significant differences were identified between the study groups regarding incidence of AKI, but statistically significant differences were shown for acute respiratory distress syndrome and multiple organ dysfunction [77]. Albreiki et al. [78], in a similar meta-analysis on fluid resuscitation with permissive hypotension in trauma patients with hemorrhagic shock, concluded that the strategy has a favorable impact on both mortality and recovery [78].

The main fluid agents currently used in the resuscitation of hemorrhagic shock, including crystalloids, colloids, and blood products, are summarized in Table 1, together with their composition, volume expansion properties, principal adverse effects, and current guideline status.

## 5. Multimodal Monitoring for Individualized Fluid Resuscitation

The cellular and molecular derangements described in the preceding sections produce physiological disturbances that can be measured at the bedside, although not all of them directly. Mitochondrial dysfunction and cytokine activation are not monitored in real time; rather, their downstream consequences, including tissue hypoperfusion, metabolic acidosis, impaired coagulation, and reduced peripheral oxygenation, are what clinical monitoring captures. These parameters are most informative when interpreted together and followed over time rather than against any single fixed threshold, allowing resuscitation to be titrated to the individual patient’s evolving physiology. The following subsections describe the biological rationale and clinical evidence for each monitoring domain.

### 5.1. Metabolic and Perfusion Endpoints

Serum lactate concentration and lactate clearance are among the most widely validated metabolic endpoints in the management of hemorrhagic shock. Elevated lactate reflects the shift toward anaerobic metabolism secondary to inadequate tissue oxygen delivery, and serial lactate measurements provide a dynamic picture of resuscitation adequacy. A lactate clearance of at least 10% per hour has been associated with improved survival outcomes in critically ill patients [79], and failure to normalize lactate within the first 6–12 h after trauma is a strong predictor of multiple organ dysfunction and mortality [80]. The combination of serum lactate and shock index (heart rate divided by systolic blood pressure) has been shown to improve the predictive accuracy for massive transfusion requirements, with combined thresholds of lactate greater than 4 mmol/L and shock index above 1 yielding a specificity exceeding 95% for massive transfusion need [81].

Base excess and arterial pH derived from blood gas analysis provide complementary information on the degree of metabolic acidosis and its correction over time. A base excess more negative than −6 mEq/L has been incorporated into consensus definitions of the polytrauma patient as an indicator of physiological derangement [79]. The normalization of base excess during resuscitation correlates with improved tissue perfusion and reduced transfusion requirements. Venous-to-arterial carbon dioxide difference (PvaCO_2_ gap) represents another sensitive indicator of the adequacy of cardiac output relative to metabolic demand; a PvaCO_2_ gap exceeding 6 mmHg in the context of a normal central venous oxygen saturation suggests residual low-flow states even after apparent hemodynamic stabilization, and should prompt further resuscitation or vasoactive support [82].

### 5.2. Dynamic Hemodynamic Parameters and Fluid Responsiveness

Static hemodynamic parameters such as central venous pressure have been shown to be poor predictors of fluid responsiveness and have largely been replaced by dynamic preload indices in contemporary critical care practice. Pulse pressure variation (PPV) and stroke volume variation (SVV), derived from arterial waveform analysis in mechanically ventilated patients with sinus rhythm, reliably identify patients who will respond to a fluid challenge with a significant increase in cardiac output. A PPV or SVV threshold above 12–13% is generally considered predictive of fluid responsiveness [83]. These parameters can be continuously monitored using minimally invasive arterial waveform analysis devices or via advanced hemodynamic monitoring platforms such as PiCCO (Pulse Contour Cardiac Output), which additionally provides thermodilution-derived measurements of cardiac index, global end-diastolic volume index, and extravascular lung water index. The latter is particularly relevant in the polytrauma context, as it allows the early detection of pulmonary fluid overload before it becomes clinically apparent, thereby enabling a more precise titration of fluid therapy [84].

Point-of-care ultrasonography (POCUS) has become an indispensable bedside tool in the emergency and critical care assessment of polytrauma patients. Focused assessment with sonography in trauma (FAST) and its extended variant (eFAST) provide rapid identification of hemopericardium, pneumothorax, and intra-abdominal free fluid, directly informing resuscitation priorities. Additionally, inferior vena cava (IVC) collapsibility index assessed by transthoracic ultrasonography offers a semiquantitative estimate of volume status and preload responsiveness [85], particularly useful in spontaneously breathing patients in whom PPV and SVV are unreliable. Left ventricular function and wall motion abnormalities can also be rapidly screened, allowing differentiation of hemorrhagic from cardiogenic shock contributions in complex polytrauma scenarios [86].

### 5.3. Point-of-Care Viscoelastic Coagulation Testing

Trauma-induced coagulopathy (TIC) is a frequent and life-threatening complication in hemorrhagic shock, present in up to 25–35% of severely injured patients on hospital admission [87]. Standard coagulation tests (prothrombin time, activated partial thromboplastin time, international normalized ratio) provide limited information on the dynamic and often rapidly evolving coagulation derangements seen in TIC, and their turnaround time is poorly suited to real-time resuscitation guidance. Viscoelastic hemostatic assays, including thromboelastography (TEG; Haemonetics Corporation, Boston, MA, USA) and rotational thromboelastometry (ROTEM; Werfen/Instrumentation Laboratory, Bedford, MA, USA), overcome these limitations by providing a comprehensive, fast, although not truly real-time assessment of the entire coagulation process from clot initiation to fibrinolysis [88]. These assays allow rapid identification of specific coagulation defects, including hyperfibrinolysis, fibrinogen deficiency, thrombocytopenia, and factor deficits, enabling targeted rather than empirical blood product administration. Multiple studies and international trauma guidelines have endorsed TEG- and ROTEM-guided resuscitation protocols as superior to conventional coagulation-based protocols in reducing blood product use and improving survival in massive hemorrhage [89,90]. The outcome evidence for this individualized approach is mixed. In a single-center randomized trial of 111 patients meeting massive-transfusion criteria, a protocol goal-directed by TEG reduced 28-day mortality compared with one guided by conventional coagulation assays (19.6% vs. 36.4%; *p* = 0.049) while using less plasma and fewer platelets in the early phase [91]. The multicenter ITACTIC trial did not confirm an overall survival advantage of viscoelastic-guided over conventional-test-guided major-hemorrhage protocols (28-day mortality 25% vs. 28%), plausibly because the coagulopathy encountered was milder than anticipated and both arms already operated within empirical major-hemorrhage protocols; a mortality benefit did, however, emerge in the prespecified subgroup with traumatic brain injury [89]. Viscoelastic guidance is therefore most valuable where coagulopathy is established and evolving, rather than as a universal replacement for protocolized hemostatic resuscitation. A key therapeutic application of viscoelastic hyperfibrinolysis detection is the targeted use of the antifibrinolytic agent tranexamic acid (TXA), a synthetic lysine analogue that competitively inhibits plasminogen activation and thereby stabilizes formed clot. The CRASH-2 trial demonstrated that early TXA administration (within 3 h of injury) significantly reduced all-cause mortality and death due to bleeding in trauma patients, with no increase in vascular occlusive events, establishing it as a cornerstone of hemostatic resuscitation [92]. The biological rationale for TXA is reinforced by viscoelastic testing, which can identify the subset of patients with demonstrable hyperfibrinolysis who derive the greatest benefit, while also cautioning against indiscriminate use in patients exhibiting “fibrinolysis shutdown”, a paradoxical hypofibrinolytic state associated with its own adverse outcomes [10]. This illustrates the broader principle of the monitoring framework: a single molecular endpoint, the fibrinolytic state, directly determines a specific, individualized therapeutic decision. The integration of point-of-care viscoelastic testing into trauma resuscitation algorithms therefore represents a key component of the multimodal monitoring strategy, directly informing decisions on fibrinogen concentrate, cryoprecipitate, platelet, and fresh frozen plasma administration.

### 5.4. Near-Infrared Spectroscopy and Tissue Oxygenation Monitoring

Near-infrared spectroscopy (NIRS) enables continuous, non-invasive monitoring of regional tissue oxygen saturation (rSO_2_) at the microvascular level, most commonly applied to the thenar eminence or the deltoid muscle. In hemorrhagic shock, peripheral rSO_2_ values fall early and reflect the redistribution of blood flow away from non-vital tissue beds, making NIRS a sensitive early warning indicator of hemodynamic decompensation preceding changes in systemic blood pressure or heart rate [93]. The dynamic vascular occlusion test (VOT), performed in conjunction with NIRS, evaluates microvascular reactivity and the rate of rSO_2_ recovery after a brief period of ischemia, providing information on the functional reserve of microcirculation. Impaired VOT recovery rates have been associated with worse outcomes and greater transfusion requirements in trauma patients. Integration of NIRS monitoring into the resuscitation protocol allows titration of fluid and blood product therapy to peripheral tissue perfusion rather than to systemic hemodynamic endpoints alone, supporting a more comprehensive and physiologically informed approach to individualized care [94]. The clinical utility of NIRS in this role nonetheless remains uncertain, as outcome data specific to hemorrhagic shock are limited. Its rationale is best considered alongside the broader question of whether targeting peripheral perfusion improves outcomes. In septic shock, the ANDROMEDA-SHOCK trial compared resuscitation targeted to peripheral perfusion, assessed by capillary refill time, with a lactate-guided strategy and found no significant difference in 28-day mortality (34.9% versus 43.4%; hazard ratio 0.75; *p* = 0.06), although the perfusion-targeted group had less organ dysfunction. While derived from sepsis, this result illustrates both the promise and the current limitations of perfusion-guided resuscitation, and it cautions against over-reliance on any single tissue-perfusion endpoint in hemorrhagic shock [95].

Taken together, these monitoring modalities form an integrated framework for goal-directed, individualized fluid resuscitation. No single parameter is sufficient in isolation; optimal management requires the simultaneous interpretation of metabolic endpoints (lactate, base excess, PvaCO_2_ gap), dynamic hemodynamic indices (PPV, SVV, cardiac index, extravascular lung water), bedside imaging (POCUS, IVC assessment), viscoelastic coagulation profiling (TEG/ROTEM), and tissue perfusion monitoring (NIRS). This multimodal approach enables clinicians to move beyond fixed resuscitation volumes and ratios toward a real-time, patient-specific therapeutic strategy that minimizes the dual risks of under-resuscitation and fluid overload.

**Figure 2:**
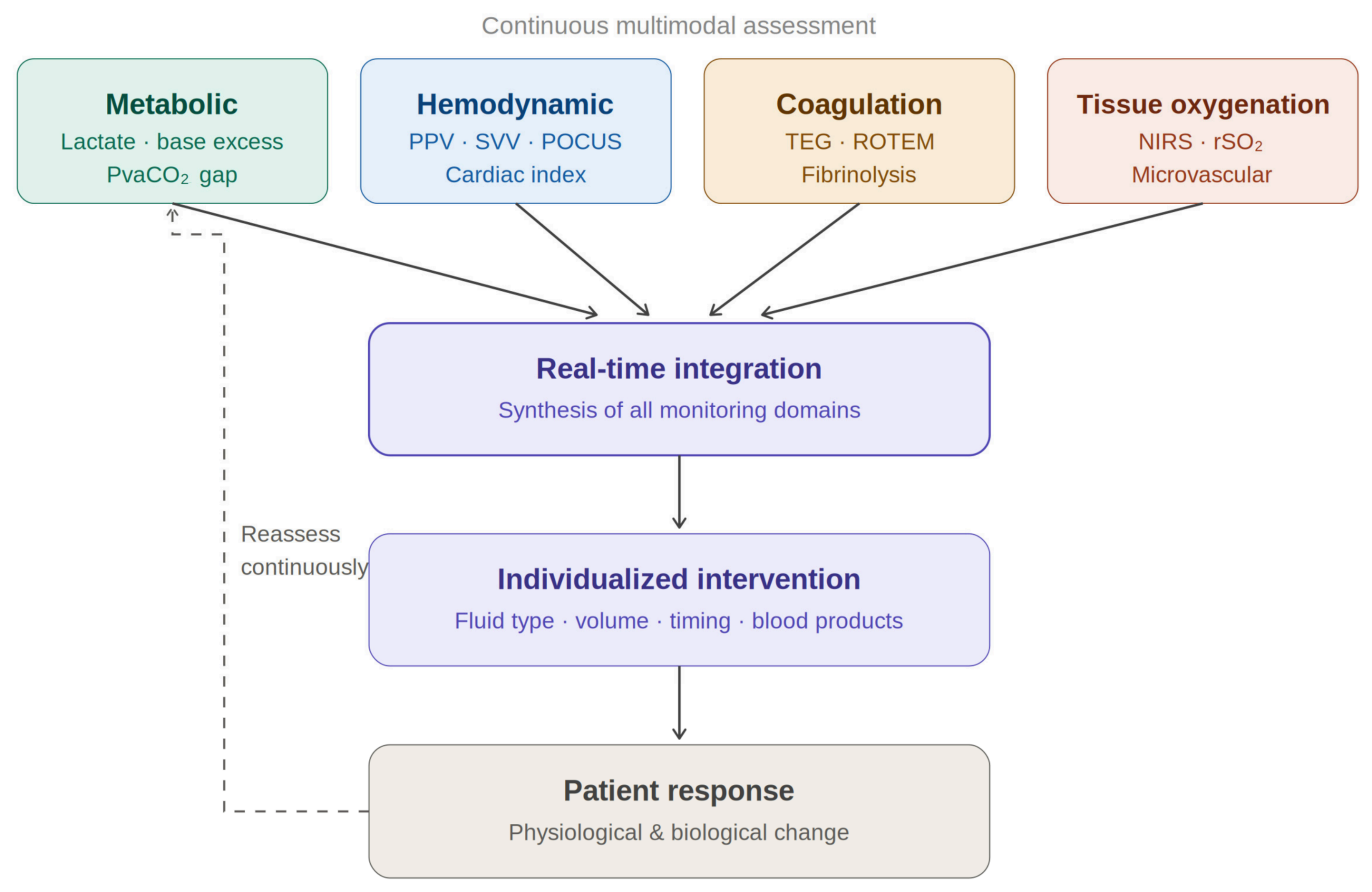
Continuous multimodal assessment for individualized goal-directed fluid resuscitation in hemorrhagic shock. Abbreviations: PvaCO_2_ = venous-to-arterial carbon dioxide difference; PPV = pulse pressure variation; SVV = stroke volume variation; POCUS = point of care ultrasound; rSO_2_ = regional tissue oxygen saturation; NIRS = near-infrared spectroscopy; TEG = Thromboelastography; ROTEM = rotational thromboelastometry. Solid arrows indicate the forward sequence of the assessment–intervention pathway; the dashed arrow denotes the continuous feedback loop by which patient response prompts ongoing reassessment.

The integration of these monitoring modalities into a unified clinical framework, encompassing metabolic endpoints, dynamic hemodynamic parameters, viscoelastic coagulation assessment, and tissue oxygenation monitoring, is illustrated in Figure 2.

## 6. Blood Transfusion in Hemorrhagic Shock

In the resuscitation of hemorrhagic shock, blood is not one fluid option among several but the definitive resuscitation fluid, because hemorrhage is fundamentally the loss of the oxygen-carrying, coagulation, and endothelial-protective functions that only blood restores. The crystalloid and colloid strategies discussed in Section 4 address intravascular volume but none of these functions, and they dilute the residual red-cell mass, coagulation factors, and platelets; they are therefore best understood as bridges that maintain perfusion during initial vascular access or when blood is not immediately available, rather than as equivalent alternatives. Modern damage-control resuscitation reflects this hierarchy, prioritizing the early, balanced delivery of blood and blood products and minimizing crystalloid [90]. The remainder of this section examines the biological rationale for each component of transfusion therapy and the evidence guiding its use, and then considers what can be done when blood itself is unavailable.

Blood transfusion and blood product administration in hemorrhagic shock represent not merely a volume replacement strategy but a direct biological intervention targeting the cellular consequences of acute hemorrhage. Red blood cell transfusion restores oxygen-carrying capacity and tissue DO_2_; plasma administration modulates coagulation factor availability, mitochondrial metabolism, and endothelial integrity; platelet transfusion supports primary hemostasis; and fibrinogen concentrate directly addresses the coagulation defect central to trauma-induced coagulopathy. Understanding the biological mechanisms underlying each of these interventions and the molecular consequences of their timing, ratio, and composition is essential for rational, individualized transfusion management in polytrauma patients with hemorrhagic shock.

In the last decade, the transfusion of blood and blood products has been widely studied and, in many countries, it is considered one of the components of national security. Blood and blood products that are currently used in clinical practice are red blood cells, platelets, and plasma. From plasma, fibrinogen concentrate and cryoprecipitate can be further obtained [96]. In trauma patients with hemorrhagic shock, a series of studies and guidelines recommend using red blood cells (RBC), platelets and plasma in different combinations and concentrations. The efficiency of RBC is given not only by the volume, but also by their age and storage [97]. Chang et al. analyzed the quality of RBC depending on the time and temperature of storage of the blood. They compared blood stored frozen to fresh blood at 7, 14, 21, 28 and 42 days. Frozen blood, in comparison with fresh blood after 7 days, had a lower number of red blood cells (3.7 vs. 5.3 × 10^6^ cells/uL, *p* < 0.01, hematocrit 33 vs. 46.5%, *p* < 0.01, hemoglobin 12 vs. 16.5 g/dL, *p* < 0.01 and pH 6.27 vs. 6.72, *p* < 0.01). The authors concluded that frozen blood loses efficiency over time and the cells become less resistant to osmotic pressure [98]. The practical implication is that red-cell transfusion restores oxygen-carrying capacity, but its benefit is tempered by storage age and transfusion-related risk, favoring fresh units and judicious triggers in massive hemorrhage. Other complications and reactions associated with transfusions have been described in the literature, such as increased systemic inflammation, risk of infection, transfusion-related lung injury (TRALI), and errors concerning administration [99]. An important trial was carried out by Holcomb et al. regarding the clinical prognosis of the mixture plasma: platelets: red blood cells 1:1:1 vs. 1:1:2 ratio in patients with severe trauma. A total of 338 patients received blood and blood products 1:1:1 during fluid resuscitation, and 342 patients received the blood products 1:1:2. Mortality rates did not differ significantly between the two groups at 24 h after resuscitation (12.7% vs. 17%, difference −4.2, 95%CI −9.6% to 1.1%, *p* = 0.12), and at 30 days after resuscitation (22.4% vs. 26.1%, difference −3.7%, 95%CI −10.2% to 2.7%, *p* = 0.26). No statistically significant differences were seen regarding secondary adverse effects associated with trauma such as multiple organ failure, sepsis, venous thromboembolism, acute respiratory distress syndrome, or other complications related to transfusion. However, the patients who received transfusion based on the 1:1:1 protocol presented with better hemodynamic stability and shorter bleeding times [100]. The message is that a balanced 1:1:1 ratio does not reduce overall mortality relative to 1:1:2 but achieves hemostasis faster and reduces death from exsanguination, supporting balanced empirical ratios until bleeding is controlled.

The choice between plasma and purified clotting factor concentrates for early hemostatic resuscitation has been directly examined in the FiiRST-2 trial, a multicenter randomized study conducted at six Canadian level I trauma centers in patients with massive hemorrhage protocol activation [101]. Patients were randomized to receive fibrinogen concentrate (4 g) plus prothrombin complex concentrate (2000 IU) or four units of frozen plasma within the initial resuscitation packs. Among the 137 patients in the primary analysis, mean 24 h transfusion requirements did not differ significantly between the factor-concentrate and plasma groups (20.8 versus 23.8 units; mean ratio 0.87; *p* = 0.20 for superiority), and no significant differences were observed in thromboembolic complications or in 24 h and 28-day mortality. The trial was terminated at interim analysis for futility. These findings indicate that, in the early resuscitation of massive traumatic hemorrhage, clotting factors are not superior to frozen plasma, and that both approaches yield comparable efficacy and safety, supporting flexibility in product selection based on availability and local logistics.

Beyond its equivalence to factor concentrates, many studies have identified the advantage of plasma administration in the initial phases of fluid resuscitation in the case of hemorrhagic shock, although different meta-analyses also reported a higher incidence of multiple organ failure. D’Alessandro et al. showed the positive impact the administration of plasma has on hypovolemic shock in lactate levels, on mitochondrial metabolism, and on protein oxidation. The author group concluded that this also has a positive impact on coagulation [102].

Makley et al. [103], also carried out an experimental study on animal models with induced hemorrhagic shock where they compared two groups of subjects, one receiving lactated Ringer’s for fluid resuscitation, and the other fresh whole blood. The IL-6, IL-10 and macrophage-derived chemokines were significantly higher in the animals receiving lactated Ringer’s compared with those in which resuscitation was based on fresh whole blood. The group that received crystalloid solutions suffered lung injury, with increased pulmonary capillary permeability. No significant differences have been noted between the study groups regarding mortality rates [103].

Seheult et al. [104] studied the clinical impact of transfusion of less than four low titer group O whole blood (LTOWB) units in trauma patients retrospectively. They included 135 patients receiving a mean of 2 units of LTOWB and 135 patients receiving conventional blood products. They found differences in mortality between the groups (24.4% vs. 18.5%, *p* = 0.24), mortality at 24 h (12.6% vs. 8.9%, *p* = 0.033%), length of ICU stay, and length of hospital stay. The time until normalization of lactate levels was also different between the two study groups, with a median of 8.1 h vs. 13.2 h, *p* = 0.05 in patients who received LTOWB [104]. A similar study was carried out by Marsden et al. on remote damage resuscitation. They administered LTOWB during transport in the pre-hospital phase and compared it with the administration of crystalloids. The study results showed a decrease in mortality for patients who benefited from the LTOWB resuscitation formula compared to conventional crystalloid resuscitation [105].

Trauma-induced coagulopathy is itself modified by the choice of transfusion strategy [106]. Spinella et al. compared warm fresh whole blood (WFWB) with stored blood components in trauma patients: one group of 100 patients received WFWB together with red blood cells and plasma, and a second group of 254 patients received red blood cells and plasma without WFWB. With comparable baseline hemoglobin, international normalized ratio, base deficit, systolic blood pressure, Injury Severity Score, and Glasgow Coma Scale, survival was higher in the WFWB group (96% vs. 88%, *p* = 0.018) [107].

Together, these findings indicate that whole blood, by delivering red cells, plasma, and platelets in physiological proportion, better preserves hemostasis than reconstitution from stored components and, given prehospital, is associated with lower mortality than crystalloid-based resuscitation, which underlies its emerging role in damage-control and remote settings. This role has expanded rapidly in recent years, with low-titer group O whole blood translating from military to civilian and prehospital practice and increasingly serving as a first-line product in major hemorrhage, although comparative outcome data continue to mature.

A similar study was conducted by Sperry et al. [108], with 501 participants with trauma associated with a risk of hemorrhagic shock, on the efficacy and clinical impact of pre-hospital administration of thawed plasma. Study participants were divided into two groups: 230 patients receiving plasma and 271 patients receiving conventional resuscitation with crystalloid and colloid solutions. Mortality at 30 days was significantly lower in patients receiving plasma during the fluid resuscitation (23.2% vs. 33%, difference −9.8%, 95%CI −18.6 to −1%, *p* = 0.03). No differences have been noted for acute respiratory distress syndrome, nosocomial infections and multiorgan failure [108]. The implication is that plasma acts not merely as a volume expander but as an endothelial and glycocalyx-restorative therapy, so that its early, even prehospital, administration can reduce mortality in patients at risk of hemorrhagic shock.

Adverse effects of hemorrhagic shock in trauma patients are related to a series of multiorgan complications that are further associated with endothelial hyperpermeability. This hyperpermeability arises principally from degradation of the endothelial glycocalyx and disruption of the interendothelial junctions, processes that impair barrier function without necessarily causing cell death; frank endothelial cell death, when it occurs, proceeds through both apoptosis and necrosis. Apoptotic signaling nonetheless contributes to endothelial injury, and Wang and He, in an experimental study on the molecular pathways involved in endothelial dysfunction associated with hemorrhagic shock, have identified an increase in the expression of CD146+ AnnexinV+ that further increases apoptosis of these cells. Animal models with induced hemorrhagic shock received fresh frozen plasma (FFP) transfusion, and have shown a decrease in the expression of pro-apoptotic cells, a decrease in vascular hyperpermeability, leading the authors to the conclusion that early plasma administration in fluid management of trauma patients with hemorrhagic shock might be beneficial [109]. These findings are consistent with the glycocalyx-protective effects discussed in Section 3.6. supporting the concept that plasma exerts its benefit not only through coagulation factor replacement but through active restoration of endothelial barrier integrity.

Curry et al. [110] conducted a randomized trial on the impact of cryoprecipitate administration in the early phases of resuscitation of trauma patients with hemorrhagic shock. There were two study groups: one group received standard treatment, and the other received cryoprecipitate in the early fluid resuscitation phase. Eighty-five percent of the patients in the study group received cryoprecipitate in the first 90 min following hospital admission, and fibrinogen values were maintained in the higher range (approx. 1.8 g/L) throughout the time of active bleeding. No statistically significant differences have been shown regarding 28-day mortality following this study [110].

Beyond the established blood products, the current research is increasingly directed at the endothelium itself. The recognition that hemorrhagic shock produces an endotheliopathy of trauma, in which early shedding of the endothelial glycocalyx drives vascular barrier breakdown, coagulopathy, and organ dysfunction, has reframed resuscitation as an opportunity not only to replace lost volume and oxygen-carrying capacity but to protect and restore the endothelial surface. Plasma, and to a lesser extent, whole blood, attenuates glycocalyx shedding and restores barrier integrity in preclinical models, an effect attributed to soluble factors that stabilize the endothelial cytoskeleton and re-establish syndecan-1 and the glycocalyx after hemorrhagic shock [111]. Building on this concept, glycocalyx-targeted and endothelial-stabilizing therapies, together with the continued optimization of plasma-based and whole-blood strategies, represent an active and hypothesis-generating direction that may in future complement conventional damage-control resuscitation. These approaches remain largely investigational, but they mark a conceptual shift from volume-centered toward endothelium-centered resuscitation [112].

Taken together, the evidence establishes a clear practical hierarchy for fluid management in hemorrhagic shock. When hemorrhage is the primary problem, blood and blood products are the definitive resuscitation, delivered early and in balanced proportion (Section 6), applied here according to the phase of injury. Purified factor concentrates are a reasonable and logistically convenient alternative to plasma where available. When blood is not immediately available, as in the prehospital or resource-limited setting, crystalloids provide a temporizing bridge that maintains perfusion while restoring neither oxygen-carrying capacity nor hemostasis, and should be limited to avoid dilutional coagulopathy and glycocalyx injury; colloids offer no advantage in this role and, in the case of starches, cause harm. This hierarchy is applied in a time-dependent manner, as set out in Section 7.

## 7. Individualized, Time-Based Resuscitation Strategy

Hemorrhagic shock is not a single event but an evolving process, and the biological threat changes with time after injury. The mechanisms described above do not operate simultaneously: complement activation, neutrophil-driven injury, and the oxidative burst dominate the early hours, whereas immune suppression, catabolism, and impaired tissue regeneration characterize the later course. Effective resuscitation therefore requires distinct, time-dependent priorities rather than a uniform strategy.

In the immediate phase, spanning the first minutes to hours, the objective is general stabilization and interruption of the lethal triad before decompensation. This phase is dominated by hemorrhage control, whether surgical, endovascular, or mechanical, and by damage-control resuscitation. Perfusion is restored to a minimally adequate rather than a normal level through permissive hypotension until bleeding is controlled, with the important exception of concomitant traumatic brain injury, where higher perfusion pressure is required. Resuscitation is hemostatic and blood-based, using balanced ratios of red cells, plasma, and platelets or whole blood, with deliberate minimization of crystalloid to avoid dilutional coagulopathy and further glycocalyx injury. When blood products are not immediately available, or during the establishment of vascular access, a balanced crystalloid such as Ringer’s lactate or Plasma-Lyte is the preferred bridging fluid, given in the smallest volume sufficient to sustain a permissive blood pressure rather than to normalize it, with isotonic saline reserved for concomitant traumatic brain injury (Section 4.4) [92].

Once hemorrhage is controlled and perfusion re-established, an intermediate phase of optimization and de-resuscitation begins, typically within the first hours to days. Here the principal threat is no longer exsanguination but ischemia-reperfusion injury: the restoration of flow to previously hypoxic tissues provokes a burst of ROS and a second inflammatory hit that amplifies the oxidative and cytokine mechanisms detailed in Section 3. No cytokine-directed therapy has proven effective in hemorrhagic shock, and the inflammatory cascade is therefore best addressed indirectly, by removing its drivers rather than by targeting the mediators themselves. Rapid hemorrhage control and restoration of perfusion limit the ischemic and complement-derived stimulus for cytokine production, and avoidance of excessive crystalloid prevents the second inflammatory hit; direct immunomodulatory approaches remain experimental. Management shifts accordingly from volume expansion to fluid stewardship, since the positive fluid balance that often follows aggressive early resuscitation independently worsens endothelial edema and organ dysfunction. Perfusion is titrated toward normal endpoints, evolving coagulopathy is monitored, and endothelial and glycocalyx integrity is actively managed. The glycocalyx is protected chiefly by avoiding the stimuli that shed it, namely prolonged hypoperfusion, hyperglycemia, and the volume overload that follows aggressive crystalloid resuscitation, and it is actively restored by early plasma or whole blood, which repair the barrier rather than merely expanding volume.

In the late phase, extending over the subsequent days, the clinical trajectory diverges. Modern early resuscitation has substantially reduced the incidence of early fulminant multiple organ failure, but a significant proportion of survivors now progress to a lingering phenotype of chronic critical illness described as persistent inflammation, immunosuppression, and catabolism syndrome (PICS). This state is driven by emergency myelopoiesis, suppression of adaptive immunity, and ongoing protein catabolism with sarcopenia, and it predisposes to recurrent nosocomial infection and indolent late death. The intestinal barrier is a central participant in this phase, since loss of gut integrity propagates systemic inflammation and helps sustain the syndrome. The therapeutic priorities here, including infection prevention, nutritional and anti-catabolic support, and emerging immunomodulatory strategies, are directed at intermediate and late cellular injury and regeneration rather than at the acute hemorrhage, and they define the biological rationale for extending individualized care well beyond the initial resuscitation [113,114].

Taken together, the evidence reviewed above indicates that individualizing resuscitation to the patient’s measured biology, rather than applying fixed volume rules, is not only mechanistically justified but is associated with improved clinical outcomes across several independent lines of evidence. The benefit is most consistent where individualization governs what is transfused and how coagulation is corrected: plasma-first and whole-blood strategies that restore endothelial and mitochondrial function reduce mortality relative to crystalloid-based resuscitation, and correcting trauma-induced coagulopathy under viscoelastic guidance rather than by conventional assays has lowered 28-day mortality in randomized comparison. The evidence is more modest, and remains largely physiological or prognostic, for the moment-to-moment titration of volume and pressure to dynamic preload, lactate clearance, or tissue-oxygenation targets, several of which are extrapolated from sepsis and are not yet validated as treatment targets in trauma. The practical implication is that the return on individualized care is greatest at the level of product selection and coagulation management, and that monitoring modalities should be weighed accordingly rather than treated as interchangeable.

## 8. Conclusions

Over the past decade, substantial progress has been made in elucidating the cellular and molecular mechanisms underlying hemorrhagic shock and its systemic consequences. The lethal triad of hypothermia, acidosis, and coagulopathy is now understood not merely as a clinical phenotype but as the convergent outcome of distinct and interacting biological pathways—including complement activation, neutrophil-mediated tissue injury, NADPH-oxidase-driven OS, cytokine cascade dysregulation, mitochondrial respiratory chain impairment, and endothelial apoptosis with degradation of the endothelial glycocalyx and junctional disruption. Each of these mechanisms represents both a therapeutic target and a source of measurable biological signals that can guide individualized resuscitation decisions.

The biological consequences of fluid administration are equally complex. Crystalloid composition directly modulates acid–base homeostasis, chloride-mediated renal vasoconstriction, coagulation factor activity, and immune cell function. Colloid oncotic properties influence plasma volume, endothelial integrity, and organ perfusion at the microvascular level. Blood products, particularly plasma and whole blood, exert active biological effects on mitochondrial metabolism, endothelial permeability, and pro-apoptotic signaling, beyond their role as volume expanders. The recognition of these mechanisms has driven a shift away from empirical, volume-based resuscitation toward biologically informed, goal-directed strategies incorporating permissive hypotension, hemostatic resuscitation, and individualized fluid selection.

Multimodal monitoring represents the translational interface between biological pathways and bedside therapeutic decisions. Metabolic endpoints such as lactate clearance and base excess reflect the adequacy of cellular oxygen delivery; dynamic hemodynamic parameters quantify preload responsiveness at the cardiovascular level; viscoelastic coagulation assays characterize the real-time biological state of the coagulation cascade; and near-infrared spectroscopy monitors microvascular oxygen utilization at the tissue level. The integration of these modalities into a unified goal-directed framework enables clinicians to move beyond fixed resuscitation protocols toward a patient-specific biological approach.

Nevertheless, significant gaps remain. No universally accepted resuscitation protocol exists that is applicable across the full spectrum of polytrauma patients, and the optimal timing, composition, and volume of fluid administration continue to vary substantially between institutions and clinical contexts. Future research should focus on several key biological questions: the molecular determinants of individual variability in the response to resuscitation fluids; the role of the glycocalyx in modulating endothelial permeability during hemorrhagic shock and its potential as a therapeutic target; the biological mechanisms underlying the survival benefit of whole blood and plasma-first resuscitation strategies; and the development of point-of-care biomarker panels that integrate genomic, proteomic, and metabolomic signals to guide truly personalized resuscitation. Advances in these areas hold the potential to transform the management of hemorrhagic shock from a predominantly empirical discipline into one grounded in precision biological medicine.

## Figures and Tables

**Table 1:** Fluid agents used in hemorrhagic shock resuscitation: composition, volume expansion efficacy, key adverse effects, and current guideline recommendations.

Agent	Composition/Type	Volume Expansion	Key Adverse Effects	Guideline Status
Crystalloids				
Normal saline 0.9%	154 mmol/L Na^+^ · 154 mmol/L Cl^−^ · 308 mOsm/L	Moderate	Hyperchloremic metabolic acidosis · renal vasoconstriction · ↓ GFR · dilution coagulopathy	Second-line crystalloid; outcomes comparable to balanced solutions but large volumes promote hyperchloremic acidosis; specific indications (e.g., hyponatremia, traumatic brain injury)
Balanced crystalloids (Ringer’s lactate, PlasmaLyte, Sterofundin)	Near-physiologic electrolytes · metabolizable anions (lactate/acetate/malate)	Moderate	Mild ↓ osmolarity (hypotonic variants) · ↑ lactate with Ringer’s in hepatic failure · slight ↑ diuresis · higher acquisition cost than saline at comparable outcomes	Recommended—first-line crystalloid in hemorrhagic shock
Hypertonic saline 7.5%	High Na^+^/Cl^−^ · 2400 mOsm/L	High (rapid)	Hypernatraemia · hyperchloremia · rebound hypovolemia · ↑ osmolarity	Selective use—TBI with raised ICP · permissive hypotension protocols
Colloids				
Human albumin 4–5%	Iso-oncotic · natural protein · long half-life	High	Cost · no survival benefit vs. crystalloid in general ICU · ↑ mortality in TBI (SAFE trial)	Selective use—hypoalbuminemia · sepsis (cautious); contraindicated in TBI
Albumin 20–25%	Hyper-oncotic · plasma volume expander	Very high	Fluid shifts · electrolyte imbalance · cost	Limited evidence in trauma; mainly hepatic/oncology use
HES 130/0.4 or 0.42	Hydroxyethyl starch · semi-synthetic · 6%	High	Acute kidney injury · osmotic nephrosis · pruritus · coagulopathy · tissue accumulation	Restricted/suspended—EMA suspension in sepsis/ICU; trauma use controversial
Gelatin (Gelaspan 4%)	Succinylated gelatin · bovine-derived · balanced	Moderate	Anaphylaxis risk · coagulopathy at high volumes · renal impairment (less than HES)	Selective use—alternative when albumin unavailable; evidence limited in trauma
Blood products				
Packed red blood cells (pRBC)	Leukoreduced · stored ≤ 42 days	High (O_2_ carrying)	Transfusion reactions · TRALI · infection risk · ↑ inflammation with older units	Recommended—Hb trigger 7–8 g/dL; prefer fresh units in massive hemorrhage
Fresh frozen plasma (FFP)	All coagulation factors · fibrinogen · anticoagulants	Moderate	TRALI · volume overload · transfusion reactions · infection risk	Recommended—1:1:1 ratio with pRBC:platelets in massive hemorrhage
Platelets	Pooled or apheresis · ABO matched preferred	Minimal	Bacterial contamination · HLA alloimmunization · TRALI · febrile reactions	Recommended—target >50 × 10^9^/L; >100 × 10^9^/L in TBI or ongoing hemorrhage
Whole blood (low-titer group O)	pRBC + FFP + platelets · 1:1:1 pre-combined	High	ABO incompatibility risk · limited availability · logistical challenges	Emerging evidence—pre-hospital and damage control; ↓ mortality vs. crystalloid
Fibrinogen concentrate/cryoprecipitate	Concentrated fibrinogen · factor XIII · vWF	Minimal	Thrombotic risk at excess dosing · viral transmission (cryo)	Recommended—fibrinogen < 1.5–2 g/L; early in massive hemorrhage per ROTEM/TEG guidance

Abbreviations: GFR = glomerular filtration rate; TBI = traumatic brain injury; ICP = intracranial pressure; HES = hydroxyethyl starch; EMA = European Medicines Agency; TRALI = transfusion-related acute lung injury; pRBC = packed red blood cells; FFP = fresh frozen plasma; vWF = von Willebrand factor; ROTEM = rotational thromboelastometry; TEG = thromboelastography; ↓, decreased; ↑, increased.

## Data Availability

No new data were created or analyzed in this study. Data sharing is not applicable to this article.

## Abbreviations

AIS	Abbreviated Injury Scale
AKI	acute kidney injury
ARDS	acute respiratory distress syndrome
ARE	antioxidant response element
BE	base excess
DAMPs	damage-associated molecular patterns
DO_2_	oxygen delivery
EMA	European Medicines Agency
FAST	Focused Assessment with Sonography for Trauma
FFP	fresh frozen plasma
GCS	Glasgow Coma Scale
GFR	glomerular filtration rate
HES	hydroxyethyl starch
HMG-1	high mobility group protein-1
HMOX1	heme oxygenase-1 gene; HO-1, heme oxygenase-1
ICP	intracranial pressure
ICU	intensive care unit
IL	interleukin
INR	international normalized ratio
ISS	Injury Severity Score
IVC	inferior vena cava
MODS	multiple organ dysfunction syndrome
MS	molar substitution
MW	molecular weight
NETs	neutrophil extracellular traps
NHE-1	sodium–hydrogen exchanger 1
NIRS	near-infrared spectroscopy
NO	nitric oxide
Nrf2	nuclear factor erythroid 2-related factor 2
O_2_^−^	superoxide anion
OS	oxidative stress
PARP	poly(ADP-ribose) polymerase
PICS	persistent inflammation, immunosuppression, and catabolism syndrome
PPV	pulse pressure variation
pRBC	packed red blood cells
PTT	partial thromboplastin time
RBC	red blood cells
ROS	reactive oxygen species
ROTEM	rotational thromboelastometry
SIRS	systemic inflammatory response syndrome
SOD1/2/3	superoxide dismutase (isoforms 1, 2, 3)
SVV	stroke volume variation
TBI	traumatic brain injury
TEG	thromboelastography
TIC	trauma-induced coagulopathy
TLR4	Toll-like receptor 4
TNF-α	tumor necrosis factor-alpha
TRALI	transfusion-related acute lung injury
TXA	tranexamic acid; V
HA	viscoelastic haemostatic assay
VO_2_	oxygen consumption
vWF	von Willebrand factor
VOT	vascular occlusion test
WFWB	warm fresh whole blood
